# Effects of Teacher Enthusiasm and Type of Text on the Motivation and Achievement of Schoolchildren

**DOI:** 10.3389/fpsyg.2022.842521

**Published:** 2022-05-30

**Authors:** Alberto Valentín, Pedro M. Mateos, Maria Mar González-Tablas, Estrella López

**Affiliations:** Faculty of Psychology, University of Salamanca, Salamanca, Spain

**Keywords:** teacher enthusiasm, type of text, intrinsic motivation, achievement, schoolchildren

## Abstract

This study aims to analyze the effects of teacher enthusiasm and type of text on student motivation and achievement. The participants were 369 elementary school students. We used four videos showing a teacher presenting two texts (narrative or descriptive) in two conditions of enthusiasm (high or neutral). A MANOVA revealed additive effects due to enthusiasm and text type on motivation and achievement, but no interaction. Mediation analyzes indicated that enthusiasm showed direct and indirect effects through motivation only for descriptive text. Therefore, the motivational mediation between teacher enthusiasm and student achievement could be especially important when the text is descriptive.

## Introduction

As education researchers, we have often asked ourselves why the comprehension or the memory of a movie, story, or theater performance is much stronger and more durable than the experience of attending a class. This may be attributable to, among other reasons, the structure of the information presented or the degree of enthusiasm with which the information is transmitted.

In the educational field, there is no commonly shared definition of teaching enthusiasm (Kunter et al., 2011; Keller et al., 2016). However, there is greater agreement that there are at least two ways to measure teacher enthusiasm (Keller et al., 2016). The first focuses on experienced enthusiasm, measured *via* the subjective feelings of the teacher (Taxer and Frenzel, 2018). The second focuses on displayed enthusiasm, measured through the observable behaviors of the teacher during the presentation of a class (Keller et al., 2014). Here, the enthusiasm displayed by the teacher can be considered their personal style of presenting information. That is, it constitutes the particular expressiveness with which the teacher transmits the educational contents. The displayed enthusiasm is usually operationalized according to Collins guidelines (Collins, 1978): fundamentally, through indicators of non-verbal behavior, such as voice intonation, facial expressions, etc.

Some empirical studies have tried to link the enthusiasm expressed by the teacher with the quality of the teaching they provide or with the students’ results (for a review of these studies, see Keller et al. (2016)). With regard to student outcomes, past research has analyzed the effects of enthusiasm on both motivation and achievement. It has consistently been shown that teacher enthusiasm has a positive effect on student motivation, defined as intrinsic motivation (Patrick et al., 2000; Frenzel et al., 2010; Keller et al., 2014; Lazarides et al., 2019), or enjoyment (Frenzel et al., 2009, 2018; Kunter et al., 2013). However, the effects of teacher enthusiasm on student achievement have not been similarly demonstrated by past research. Most studies (Bettencourt et al., 1983; Burts et al., 1985) have not found a relationship between teacher enthusiasm and student grades. Thus, Kunter et al. (2013) corroborated the effect of enthusiasm on motivation but found only some indirect effects on achievement. Hence, the relationship between teacher enthusiasm and student achievement is far from being a simple relationship.

The general theoretical framework guiding previous research on student enthusiasm and motivation has been social-cognitive learning theory (Bandura, 2001). According to this framework, the teacher acts as a model transmitting emotions and values to their students regarding the learning tasks (Frenzel et al., 2009). This is the main idea of the control-value theory of achievement emotions (Pekrun, 2006) by hypothesizing that teacher enthusiasm could generate positive emotions in the student about the subject and teaching. At the same time, teachers’ enthusiasm could reduce negative emotions, such as students’ class-related boredom, as shown in a series of studies by Cui et al. (2017, 2020, 2021).

There is, however, no clear theoretical framework to explain the direct influence of enthusiasm on performance. At the empirical level, the indirect influence of enthusiasm on achievement has been analyzed through variables such as the increase in student attention (Moè et al., 2021). An alternative that has been less explored in research is the analysis of contextual variables that might explain some conditions under which enthusiasm affects achievement.

Concerning this line of investigation, the precise relationship between teacher enthusiasm and student achievement could depend on other important variables in the educational environment. Enthusiasm could interact both with context variables (family, educational center, etc.) and with the characteristics of the study material. Concerning these characteristics, one that has proved to be important is the structure of the study material itself. There are a large number of different types of text but they can be reduced to a more parsimonious distinction between descriptive and narrative texts (Grabe, 2002), each with its own structure of discourse.

Narrative texts use chronological order as primary form of discourse organization (beginning, development, and end). This type of discourse helps the organization of knowledge and supports student progress in understanding the context through the reality reflected in its contents (Kintsch and Young, 1984). In contrast, descriptive texts use a wide variety of discourse organizers including, among others, the enumeration of the characteristics of a person or thing, cause/effect relationships, the presentation of a problem and its solution, and the comparison or contrast between alternatives. Therefore, the descriptive structure provides the information in its entirety, in addition to conveying ideas with accuracy. However, understanding a descriptive text requires prior knowledge (Larrañaga and Yubero, 2015; López and Fernández, 2016). Thus, we can speak of narrative texts and descriptive texts as a function of the type of structure prevailing within them. Furthermore, descriptive and narrative texts differ from each other in their objectives of informing and entertaining, respectively. In short, the first highlights “what is happening,” while the second highlights “how it is happening.”

These structural differences between narrative and descriptive text could have a special impact on comprehension or recall of information and, ultimately, on student achievement (Van Dijk, 1977; Graesser et al., 1994; Álvarez-Angulo, 1996; López-Escribano et al., 2013). Thus, the use of logical connectors in the language presented, such as “In this way,” “First of all,” etc., facilitates the understanding of descriptive-expository text (Graesser and Goodman, 1985; Sánchez and García, 2009), while the logic of the sequencing of narrated events facilitates the understanding of narrative texts. This narrative logic helps one to fill in the gaps that appear in the different sequences of the narrative and to make inferences about the representation of those events (Gárate, 1994).

One possible advantage of narrative texts is that they usually entertain, or are designed to entertain (Ministerio de Educación, Cultura y Deporte, 2013, 2017). That is, narrative texts aimed at children seem to generate enjoyment or motivation (Brewer and Lichtenstein, 1982). In this way, these texts can produce an additional, affective representation of the events, making them more memorable (Gernsbacher et al., 1992). Indeed, a narrative text is memorable if it manages to entertain (Dudukovic et al., 2004). Thus, narrative texts might increase both motivation and achievement (Pham and Sánchez, 2019) in schoolchildren.

Since both teacher enthusiasm and type of text seem to affect student motivation and achievement, it is possible that the two variables could combine their effects. To our knowledge, only Moè (2016) has considered the joint effect of these two variables; however, this study does not present statistical data on the interaction. Thus, the possible interaction between enthusiasm and text type remains unclear. The present study aims to explicitly analyze such an interaction. We consider that the effects of teacher enthusiasm on achievement could be more pronounced in narrative texts than in descriptive texts. That is, teacher enthusiasm could adapt well to the structure of a narrative text. Since the comprehension of a narrative text depends mainly on the continuity of its argument, the teacher’s enthusiasm could highlight this. Conversely, we believe that teacher enthusiasm is less suited to descriptive texts. Understanding descriptive texts relies mainly on the use of logical connectors in the language, hence teacher enthusiasm could have a lower impact.

In sum, the aim of this paper is to analyze the effects of teacher enthusiasm and text type on student motivation and achievement. Specifically, our hypotheses are: (a) the high-enthusiasm condition will have a more beneficial effect on intrinsic motivation and achievement than the neutral enthusiasm condition; (b) the narrative text will have a more beneficial effect on intrinsic motivation and achievement than the descriptive text; and (c) we expect the beneficial effect of high teacher enthusiasm on intrinsic motivation and achievement to be more pronounced for the narrative text than for the descriptive text.

## Materials and Methods

### Design

Our research used an analytical, prospective, cross-sectional, and mixed factorial design, in which the enthusiasm displayed by the teacher was the between factor, and the type of text used was the within factor. Displayed enthusiasm was operationalized in two categories: high enthusiasm and neutral enthusiasm. Low enthusiasm was not incorporated into the design because, in daily teaching practice, it is not a condition opposite to high enthusiasm. For its part, type of text was operationalized into two categories (narrative and descriptive), chosen for being the most used textual modalities in teaching practice.

### Participants

The participants were 369 children, of whom 185 were males, aged between 9 and 12 years (*M* = 10.94, SD = 0.80). The children came from 32 primary education centers: public (68.9%), subsidized (21.8%), and private (9.3%). These centers were located in the autonomous communities of Castilla y León and Extremadura (Spain). The number of children from each center ranged from a minimum of 8 to a maximum of 26.

### Instruments

#### Description of the Independent Variables

We selected two texts representing the narrative and descriptive modalities. As a narrative text, the story “The Lion and the Puppy” by León Tolstoy was selected, taken from The Best Stories for Children, and translated from Russian by Bibicharifa Jakimzianova and Jorge Saura (2015). The descriptive text selected was “Bees,” taken from Hum Sweet Hum (OECD, 2009). A more detailed description of the texts used can be found in Appendix.

We made a total of 16 video recordings that showed the same teacher reading the narrative text or the descriptive text. For half of the recordings of each type of text, the teacher showed high enthusiasm, and for the other half she showed neutral enthusiasm. We selected, by agreement among judges (five expert researchers in emotional expression), the four recordings in which the teacher best reflected these levels of enthusiasm. The judgments for both levels of enthusiasm were made on the basis of the displayed enthusiasm criteria described by Collins (1978) and Murray (1983): intonation, expressivity of the eyes, gesticulation, body mobility, facial expression, and dynamism. The videos selected in this way operationally define our conditions of high and neutral enthusiasm.

We controlled several indicators of both the texts and their recordings. As regards the texts, they were similar in terms of length after eliminating articles, prepositions, conjunctions, adverbs, and repeated words. There were 106 words in the descriptive text and 121 words in the narrative text. In addition, according to the indexes collected in LexEsp (Sebastián et al., 2000), the two texts were similar in terms of the frequency of word usage [*t*(205) = −0.23, *p* > 0.819]. Other lexical indices revealed that the words in the narrative text presented greater familiarity [*t*(213) = 3.82, *p* < 0.001], concreteness [*t*(216) = 3.67, *p* < 0.001], and imaginativeness [*t*(218) = 4.09, *p* < 0.001] than the words in the descriptive text. The recordings of the descriptive text last for 2.51 min (high enthusiasm) and 2.45 min (neutral enthusiasm), whereas the narrative text has a duration of 2.49 min in both enthusiasm conditions.

#### Measurement of Dependent Variables

We measured intrinsic motivation using four questions related to the following dimensions: enjoyment, interest, pleasure, and curiosity. We constructed the questions from the wording used by Moè (2016). A 5-point Likert-type scale was used. The total score on intrinsic motivation was obtained from the average of the scores on the four questions.

In addition, we consider the estimated duration of the recording as an indirect indicator of intrinsic motivation insofar as a time estimation less than the actual time would reflect less boredom and/or greater intrinsic motivation. As such, we asked the participants to judge the time duration of each presentation using a Visual Analog Scale (0–5 min). In order to compare the time estimates attributed to each type of text, we transformed all of the scores into a single scale.

We measured achievement in terms of reading competencies (strategies and skills), as defined in the PISA Evaluation Report (2007) (OCDE/MEC, 2007). So, for each text, we evaluated: broad understanding (identifying the main idea of the text), retrieval (locating one or more fragments of information), induction (the ability to make inferences), and interpretation (the ability to extract explicit information from the text). Furthermore, we add an item to evaluate free recall (unassisted retrieval of words). A description of the items used to measure achievement, as well as how they are scored, can be found in Appendix. The score for each competency was transformed into a ratio scale. The overall achievement score was the sum of these ratio scales (with a range from 0 to 5).

To confirm the effectiveness of the teacher enthusiasm manipulation, we included a question in which the participants were asked about the degree of enthusiasm they perceived in the teacher. The response scale ranged from 0 to 5. The response of the participants constitutes our measure of the perceived enthusiasm variable.

### Procedure

A total of 32 primary education centers agreed to collaborate in the study. Prior informed consent of the parents was required in order for students to participate. In each center, two groups were selected at random, with the only condition being that in each group there was the same number of boys as girls. The information was collected during school hours, in classrooms facilitated by the centers.

The participants were informed about the task, and instructions emphasized that they should pay close attention because they would later be asked questions related to the task. Each child saw two recordings: one for the descriptive text and one for the narrative text. The order in which the recordings were presented was randomized among the participants. In addition, a perceptual judgment distracting task was used to separate the two recordings. Half of the children saw the recordings in the high-enthusiasm condition and the other half saw the recordings in the neutral-enthusiasm condition.

## Results

### Preliminary Analyses

The means and standard deviations of the main variables are shown in Table 1.

**TABLE 1:** Descriptive data of the variables.

		Type of text						
		Narrative		Descriptive		Global		
Measures	Enthusiasm	Mean	SD	Mean	SD	Mean	SD	*N*
Intrinsic	Neutral	2.89	1.04	2.30	0.88	2.59	0.80	183
motivation	High	3.39	0.88	2.83	0.88	3.11	0.71	186
	Total	3.14	1.00	2.57	0.91	2.85	0.80	369
Estimated	Neutral	2.98	1.19	3.36	1.14	3.20	0.96	183
time	High	2.31	1.03	2.81	0.94	2.55	0.79	186
	Total	2.64	1.16	3.08	1.08	2.87	0.93	369
Achievement	Neutral	1.55	0.93	1.31	1.01	1.42	0.82	183
	High	2.07	0.96	1.88	1.03	1.99	0.90	186
	Total	1.81	0.98	1.60	1.06	1.71	0.91	369

We conducted a reliability analysis of the indicators of intrinsic motivation (Cronbach’s alpha = 0.85) and achievement (Cronbach’s alpha = 0.72), with both measures showing good internal consistency. In addition, to check the effectiveness of the enthusiasm manipulation we carried out a *t*-test for the means difference on the perceived enthusiasm variable (*t* = 15.39, df = 357.58, *p* < 0.001). The result confirms that the high enthusiasm group (*M* = 3.51, SD = 0.72) perceived greater enthusiasm in the teacher’s presentation than the neutral enthusiasm group (*M* = 2.26, SD = 0.84).

### Data Analysis

To test our hypothesis, a 2 × 2 multivariate analysis was carried out, with a within factor (type of text) and a between factor (teacher enthusiasm), using intrinsic motivation, estimated time, and achievement as dependent variables. The multivariate tests were significant for both teacher enthusiasm *F*(3, 365) = 31.75, *p* < 0.001, partial η^2^ = 0.21, and type of text *F*(3, 365) = 46.44, *p* < 0.001, partial η^2^ = 0.28. No significant interaction effects were found, *F*(3, 365) = 0.40, *p* > 0.756, partial η^2^ = 0.003.

**FIGURE 1:**
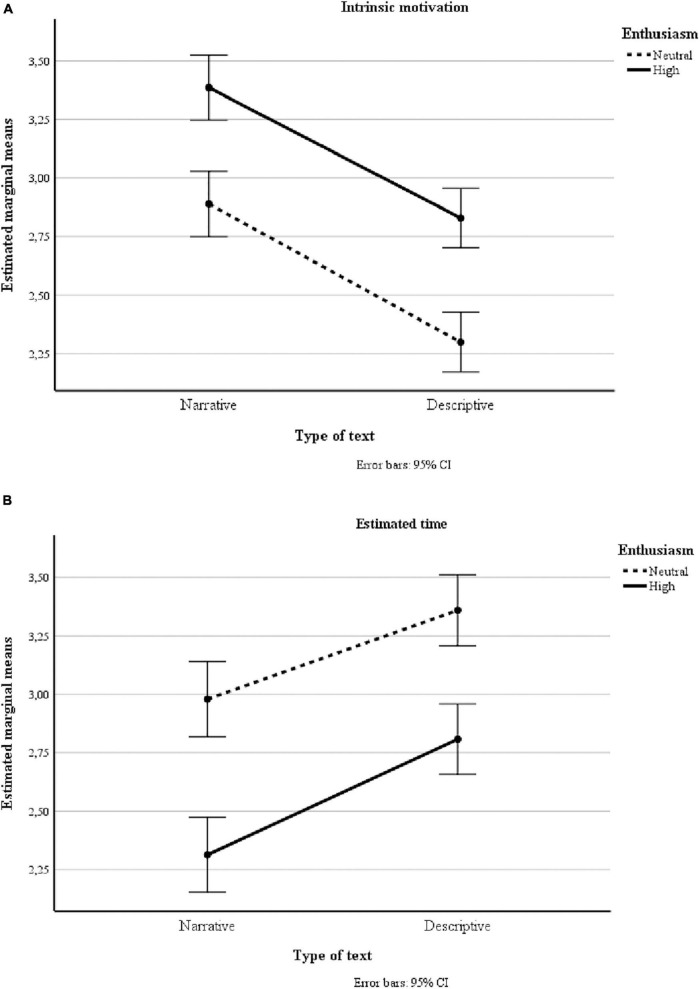
Estimated marginal means for intrinsic motivation **(A)** and estimated time **(B)** by teacher enthusiasm and type of text.

When we analyzed the effect of enthusiasm on the direct measure of intrinsic motivation (Figure 1A), the result was significant, *F*(1, 367) = 42.48, *p* < 0.001, partial η^2^ = 0.10. Scores were higher in the high enthusiasm group than the neutral enthusiasm group. Additionally, in respect of intrinsic motivation scale scores, type of text effects were found, *F*(1, 367) = 108.95, *p* < 0.001, partial η^2^ = 0.23. The narrative text scores were higher than the descriptive text scores. There were no interaction effects.

In analyzing estimated time (Figure 1B) as an indirect measure of intrinsic motivation, effects were found due to teacher enthusiasm, *F*(1, 367) = 44.68, *p* < 0.001, partial η^2^ = 0.11. The estimated time was less in the high enthusiasm group than in the neutral enthusiasm group. In addition, effects due to type of text were found, *F*(1, 367) = 44.37, *p* < 0.001, partial η^2^ = 0.11. The estimated time scores were lower for narrative text than those for the descriptive text. No interaction effects were found.

**FIGURE 2:**
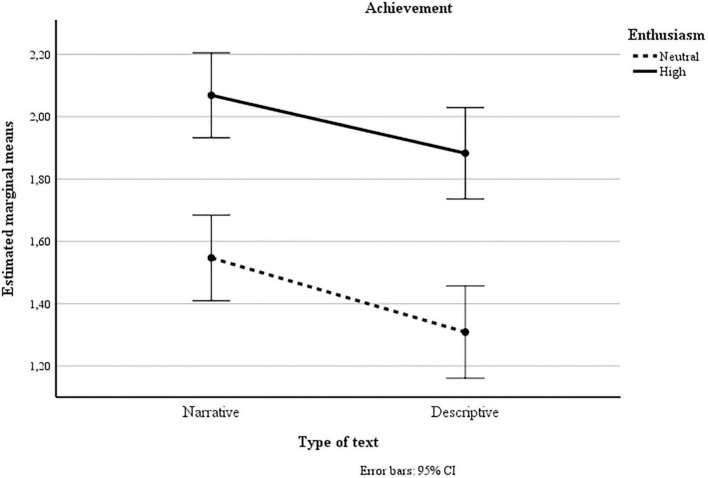
Estimated marginal means for achievement, by teacher enthusiasm and type of text.

Finally, in analyzing the measure of achievement (Figure 2), we found that the enthusiasm effects were significant *F*(1, 367) = 37.86, *p* < 0.001, partial η^2^ = 0.09. The achievement scores were higher in the high-enthusiasm group than in the neutral enthusiasm group. In addition, effects due to type of text were found, *F*(1, 367) = 17.80, *p* < 0.001, partial η^2^ = 0.05. The achievement scores were higher for the narrative text than the descriptive text. There were no interaction effects.

### Mediational Analysis

We carried out further analyses, in addition to those directly related to the hypotheses, in order to check for possible mediating effects between our study variables, using the PROCESS macro (version 2.15) for SPSS (model 4) (Hayes, 2017). We tested three multiple mediation models, in which the independent variable was teaching enthusiasm (X), the dependent variable was achievement (Y), and the mediating variables were intrinsic motivation (M1) and estimated time (M2). In the first model we used the global scores for these variables, while in the second and third models we used the scores relative to the descriptive and narrative texts, respectively.

**FIGURE 3:**
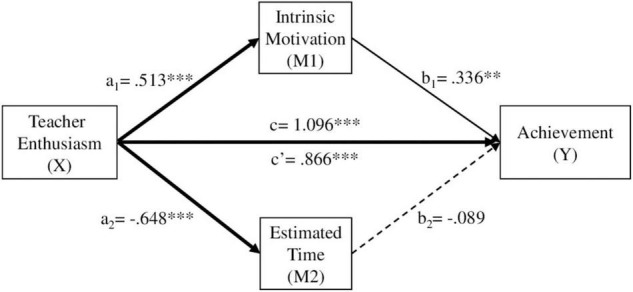
Analysis of mediation through intrinsic motivation and estimated time as a function of teacher enthusiasm over achievement. a_1_, a_2_, b_1_, b_2_, c’ = path coefficient (unstandardized coefficient). *p < 0.05, **p < 0.01, ***p < 0.001. Direct effects B = 0.866, SE = 0.194, *t* = 4.475, *p* < 0.0000, 95% CI [0.485, 1.246]. Indirect effects B = 0.230, SE = 0.084, 95% CI [0.070, 0. 401]. Total effects B = 1.096, SE = 0.178, *t* = 6.153, *p* < 0.0000, 95% CI [0.746, 1.446].

In the first model (Figure 3), the results showed statistically significant total effects for teacher enthusiasm on student achievement (c). Similarly, the direct effects of teacher enthusiasm on intrinsic motivation (a1), on estimated time (a2), and on achievement (c′) were significant. The direct effect of intrinsic motivation on achievement was also significant (b1). However, the direct effect of estimated time on achievement was not significant (b2). Regarding the indirect effects of teacher enthusiasm on student achievement, the mediation of intrinsic motivation (a1b1) was significant, but not the mediation of estimated time (a2b2).

**FIGURE 4:**
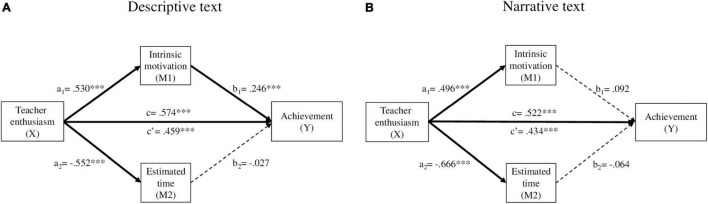
Analysis of mediation through intrinsic motivation and estimated time as a function of teacher enthusiasm over achievement: descriptive text model **(A)** and narrative text model **(B)**. a_1_, a_2_, b_1_, b_2_, c’ = path coefficient (unstandardized coefficient). *p < 0.05, **p < 0.01, ***p < 0.001. **(A)** Descriptive text: Direct effects B = 0.459, SE = 0.111, *t* = 4.151, *p* ≤ 0.000, 95% CI [0.242, 0.676]. Indirect effects B = 0.115, SE = 0.043, 95% CI [0.035, 0.204]. Total effects B = 0.574, SE = 0.106, *t* = 5.418, *p* ≤ 0.000, 95% CI [0.366, 0.782]. **(B)** Narrative text: Direct effects B = 0.434, SE = 0.104, *t* = 4.164, *p* ≤ 0.000, 95% CI [0.229, 0.638]. Indirect effects B = 0.088, SE = 0.036, 95% CI [0.022, 0.163]. Total effects B = 0.522, SE = 0.098, *t* = 5.303, *p* ≤ 0.000, 95% CI [0.328, 0.715].

In the second model (Figure 4A), we obtained results similar to those found in the first model, that is, significant direct (a1, a2, b1, and c′) and indirect (a1b1) effects. However, in the third model (Figure 4B), only the direct effects of enthusiasm on intrinsic motivation (a1), on estimated time (a2), and on achievement (c′), were significant. Thus, in this model, we do not find intrinsic motivation mediating effects on achievement.

## Discussion

Research on the effects of teacher enthusiasm on student outcomes has a long tradition. This research has consistently shown that teacher enthusiasm affects motivation, although its effects on achievement have been less clear. One way to clarify the effects of enthusiasm on achievement is to consider enthusiasm jointly with other educational context variables. In the present study, we choose a variable that we consider particularly relevant: the type of text used by the teacher.

Consistent with the results obtained in the literature (Patrick et al., 2000; Frenzel et al., 2009, 2010, 2018; Kunter et al., 2013; Keller et al., 2014; Lazarides et al., 2019), our results confirm the effect of teacher enthusiasm on the intrinsic motivation of the student. Thus, when the teacher speaks with enthusiasm, the students show greater interest in the text and the presentation time seems shorter.

Furthermore, we also found that teacher enthusiasm has beneficial effects on student achievement. This result is consistent with studies that have found an association between enthusiasm and achievement (Evertson et al., 1980; Kunter et al., 2013; Mahler et al., 2018). However, some experimental studies have failed to show that teacher enthusiasm has an effect on student achievement. These failures have led some researchers (Motz et al., 2017) to conclude that teacher enthusiasm influences student motivation, but not student achievement.

The fact that we have obtained results that differ from those of other researchers could be attributed to a bad manipulation of enthusiasm and/or an inadequate achievement measurement on our part. However, we do not believe that this was the case. On the one hand, our enthusiasm manipulation was effective. That is, the students unequivocally perceived when the teacher was expressing herself enthusiastically. In addition, to measure achievement we used the PISA Report evaluation criteria (OCDE/MEC, 2007). That is, we used an institutionally standardized procedure to measure achievement.

This discrepancy between our results and those of other researchers could be attributed to the age of the participants in our study. It is possible that enthusiasm expressed by the teacher influences learning/achievement only at the primary education stage. Perhaps the supposed effect of *emotional contagion* from the teacher (Mahler et al., 2018) only translates into greater achievement in the case of primary-age children. Like us, other authors have found effects from teacher enthusiasm on achievement in samples of primary-age children (Moè, 2016). However, contrary to this argument, McKinney and colleagues, in a wide range of studies with children of different ages (Larkins and McKinney, 1982; McKinney et al., 1984; Burts et al., 1985) found no differences attributable to age.

With regard to the type of text, we found that it affects the intrinsic motivation of the student. The narrative texts generated more intrinsic motivation than the descriptive texts, both in terms of interest and estimated time. This result was expected since narrative texts, unlike descriptive texts, are usually constructed to entertain. The narrative text that we used in this research is intended to entertain, as it is a fictitious narrative text, more than an informative narrative text (Dudukovic et al., 2004; Ministerio de Educación, Cultura y Deporte, 2013, 2017).

Similarly, we found that the type of text affects student achievement. The children obtained higher achievement on the narrative text than on the descriptive text. This may be because narrative texts, unlike descriptive texts, can add additional emotional representation to the content, making it more memorable (Gernsbacher et al., 1992; Dudukovic et al., 2004). This is in accordance with the fact that, as we point out above, the narrative text generated more motivation than the descriptive one. The superiority of the narrative text could also be due to the structure of this type of text. In this regard, the chronological presentation of events, typical of the narrative structure, might be more understandable for schoolchildren than a presentation supported by logical connectors, typical of the descriptive structure. Finally, narrative texts do not require the prior knowledge that is required for descriptive texts (Larrañaga and Yubero, 2015; López and Fernández, 2016), and this could also have contributed to the participants’ higher achievement on the narrative text.

Beyond the described effects of each variable, a central objective of our research was to analyze whether enthusiasm interacts with text type in its effects on student outcomes. With regard to achievement, contrary to our hypothesis, we were unable to confirm interaction between the degree of enthusiasm and type of text. In other words, we did not confirm that the beneficial effect of teacher enthusiasm on achievement was more pronounced in relation to narrative text than it was in relation to descriptive text. Our results suggest that both the adaptation of study material to narrative-type structures and the enthusiasm of the teacher’s presentation positively but independently affect the achievement of primary school students.

As regards motivation, we were also unable to confirm our hypothesis of an interaction between enthusiasm and type of text. That is, high motivation caused by high teacher enthusiasm was not more pronounced for the narrative text than for the descriptive one. This result was similar for the two intrinsic motivation indicators that we used: the scale of intrinsic motivation and the estimation of the time duration for each type of text. In short, we have not been able to clarify the type of text for which teacher enthusiasm exerts the most beneficial effect on student outcomes. In the present study, enthusiasm and the type of text jointly effect achievement and motivation.

The influence of teacher enthusiasm on both motivation and achievement, found in this study, leaves open the possibility that motivation acts as a mediating variable between teacher enthusiasm and student achievement. The mediation between these variables would seem to be implicit in how many educators understand the educational process. In this regard, the specialized literature suggests that one of the mechanisms by which enthusiasm influences academic achievement is precisely by fostering student motivation (Babad, 2007; Keller et al., 2013). Consequently, many authors have expressly stressed the need to empirically prove such influence (Kunter et al., 2011; Keller et al., 2016). However, some researchers claim to have shown that enthusiasm affects motivation but not achievement (Motz et al., 2017), leading them to question the mediating role of motivation. In the present research, we have attempted to demonstrate empirically, through a mediational analysis, both the direct and indirect effects of enthusiasm on achievement.

This mediational analysis provides support for the mediating role of motivation between teacher enthusiasm and student achievement. Our results suggest that when the teacher acts enthusiastically, it promotes intrinsic motivation in students and this, in turn, boosts their achievement. At the same time, the teacher’s enthusiasm makes the student’s experience of time seem shorter. However, this other form of motivation does not produce higher student achievement. Estimated time duration, as an indirect measure of motivation, is probably too generic and too distal to predict achievement.

The sequence enthusiasm–motivation–achievement, suggested by the above analysis, becomes more complex when taking into consideration the type of text. When the text is descriptive, the enthusiastic teacher encourages the students’ motivation, which, in turn, increases their achievement. When the text is narrative, the enthusiastic teacher also encourages the student’s motivation toward that text, but this motivation does not increase achievement. Therefore, motivation acts as a mediator only when the text is descriptive. That is, the characteristics of each type of text determine the mediating role of motivation. Paradoxically, narrative texts are the ones designed to entertain and, indeed, in this study the narrative text generated more intrinsic motivation than the descriptive one. Thus, the lower motivation that is associated with the descriptive text, compared to that associated with the narrative text, turns out to have a greater explanatory power in student achievement.

In conclusion, the relationship between enthusiasm–motivation–achievement suggested by our results reveals high complexity. Motivation may have more impact on achievement in less intrinsically motivating subjects. That is, teacher enthusiasm could maximize its effect on achievement when said enthusiasm is able to increase students’ interest in contents that are intrinsically less attractive.

Finally, it should be noted that we have used two different texts to operationalize the text type variable. Given the close relationship between the content of the text and its structure, it is difficult to determine the contribution of each in the effects that we have found. Future research should explore whether teacher enthusiasm might interact with textual modality when using a single text produced in two versions: one narrative and one descriptive. Similarly, it would be interesting to confirm whether our results might be generalizable among students of other ages.

## Data Availability Statement

The raw data supporting the conclusions of this article will be made available by the authors, without undue reservation.

## Ethics Statement

The studies involving human participants were reviewed and approved by Comité de Bioética from the University of Salamanca. Written informed consent to participate in this study was provided by the participants’ legal guardian/next of kin.

## Author Contributions

AV: conceptualization, project administration, funding acquisition, investigation, and writing – original draft, review, and editing. PM: conceptualization, writing – review and editing, and investigation. EL: conceptualization, visualization, and resources. MG-T: conceptualization, methodology, formal analysis, and writing – original draft. All authors contributed to the article and approved the submitted version.

## Conflict of Interest

The authors declare that the research was conducted in the absence of any commercial or financial relationships that could be construed as a potential conflict of interest.

## Publisher’s Note

All claims expressed in this article are solely those of the authors and do not necessarily represent those of their affiliated organizations, or those of the publisher, the editors and the reviewers. Any product that may be evaluated in this article, or claim that may be made by its manufacturer, is not guaranteed or endorsed by the publisher.

## Appendix

**Achievement assessment**

Two texts and a set of questions were used to measure achievement.

**Texts**

We used a descriptive text and a narrative text. The selected descriptive text is one of the texts used by PISA to assess the reading literacy of students. It is a descriptive-explanatory text called “Bees” (see https://www.oecd.org/pisa/pisaproducts/Take%20the%20test%20e%20book.pdf; pp. 56–57). Specifically, we use the adaptation of this text made for its application to Spanish schoolchildren. Regarding the narrative text, we reject the text proposed by PISA (the story by León Tolstoy “A just judge”), as it is longer and more complex than the descriptive text “Bees.” We selected instead a story, also by Leon Tolstoy, entitled “The Lion and the Puppy.” This story is a narrative text of similar length and complexity to the descriptive text. The English-speaking reader can find several editions of this story. For example, “The lion and the puppy: and other stories for children” (https://cmc.marmot.org/Hoopla/MWT12358619).

**Questions**

We evaluated the achievement of the students by means of five questions relating to each of the texts. For the descriptive text, four of these questions correspond to those used as sample questions in the PISA tests (see https://www.oecd.org/pisa/pisaproducts/Take%20the%20test%20e%20book.pdf; p. 57). We also used the scoring criteria of the PISA tests to evaluate the answers to these questions (see pp. 94–96). For the narrative text, we developed four original questions. These questions were designed to be comparable to those of the descriptive text in terms of the cognitive aspects assessed, the formats, and the order of item presentation. An English translation of the questions used for the narrative text is presented below.

Question 1

What is the purpose of providing dogs and cats to zoo animals?

A. To feed the wild animals.

B. To make friends with the wild animals.

C. To keep the dogs and cats until their owner comes to pick them up.

D. To go inside to see the wild animals.

Score 1: Choice D.

Score 0: Other options.

To answer this question correctly, students had to identify the main idea of the text.

Question 2

List three actions by the lion when the puppy turned on its back and started wagging its tail.

1. __________________________________________________________________________
2. __________________________________________________________________________
3. __________________________________________________________________________

Score 1: Three of the following actions in any order: “The lion touched him with his claw”; “The lion examined him”; “The lion moved his head”; “The lion did not hurt him”; “The lion walked away.”

Score 0: Other actions.

To answer this question correctly students had to find one or more fragments of information.

Question 3

What is the main difference between the first puppy and the second one?

A. The second puppy was eaten by the lion.

B. The first puppy was eaten by the lion.

C. The first puppy died of grief.

D. The second puppy got sick.

Score 1: Choice A.

Score 0: Other options.

To answer this question correctly students had to make inferences.

Question 4

¿Why did the lion become sad, sniff the puppy and lick it?

__________________________________________________________________________

__________________________________________________________________________

__________________________________________________________________________

Score 2: Answers that indicate both the friendship between the puppy and the lion and the lion’s grief. For example: “Because the puppy was his friend and he was sorry that the puppy died.”

Score 1: Answers that mention only one of the options. For example: “Because the puppy was his friend” or “Because he was sorry that the puppy died.”

Score 0: Irrelevant, inaccurate, incomplete, or vague responses. For example: “Because he was hungry,” “Because he wanted to play,” etc.

To answer this question correctly, students had to extract explicit information from the text.

To complete the evaluation of student achievement, we developed the following free recall question. This fifth question was applied to both texts.

Question 5

Write down everything you remember from the text. Please be as accurate as possible. If you are able to write the same words as you have heard, then that is even better.

_____________________________________________________________________________

_____________________________________________________________________________

_____________________________________________________________________________

_____________________________________________________________________________

_____________________________________________________________________________

_____________________________________________________________________________

_____________________________________________________________________________

_____________________________________________________________________________

_____________________________________________________________________________

Scoring: for the free recall score, we use the ratio of the number of valid recovered words to the total number of valid words in each text. We rate as valid words: nouns, adjectives, verbs, and numerical expressions. We do not rate: repetitions, variations of the noun (male/female, singular/plural), verb forms, and grammatical determiners such as pronouns, articles, etc.

To answer this question correctly the students had to recall the information without clues.
